# Autonomic nervous activity analysis based on visibility graph complex networks and skin sympathetic nerve activity

**DOI:** 10.3389/fphys.2022.1001415

**Published:** 2022-09-09

**Authors:** Zhipeng Cai, Hongyi Cheng, Yantao Xing, Feifei Chen, Yike Zhang, Chang Cui

**Affiliations:** ^1^ School of Instrument Science and Engineering, Southeast University, Nanjing, China; ^2^ Department of Cardiology, Jiangsu Province Hospital, The First Affiliated Hospital of Nanjing Medical University, Nanjing, China; ^3^ Gusu School, Nanjing Medical University, The Affiliated Suzhou Hospital of Nanjing Medical University, Suzhou, China

**Keywords:** autonomic nerve system, cerebral hemorrhage, heart rate variation, skin sympathetic nerve activity, visibility graph analysis

## Abstract

**Background:** Autonomic nerve system (ANS) plays an important role in regulating cardiovascular function and cerebrovascular function. Traditional heart rate variation (HRV) and emerging skin sympathetic nerve activity (SKNA) analyses from ultra-short-time (UST) data cannot fully reveal neural activity, thereby quantitatively reflect ANS intensity.

**Methods:** Electrocardiogram and SKNA from sixteen patients (seven cerebral hemorrhage (CH) patients and nine control group (CO) patients) were recorded using a portable device. Ten derived HRV (mean, standard deviation and root mean square difference of sinus RR intervals (NNmean, SDNN and RMSSD), ultra-low frequency (<0.003 Hz, uLF), very low frequency ([0.003 Hz, 0.04 Hz), vLF), low frequency ([0.04 Hz, 0.15 Hz), LF) and high frequency power ([0.15 Hz, 0.4 Hz), HF), ratio of LF to HF (LF/HF), the standard deviation of instantaneous beat-to-beat R-R interval variability (SD1), and approximate entropy (ApEn)) and ten visibility graph (VG) features (diameter (Dia), average node degree (aND), average shortest-path length (aSPL), clustering coefficient (CC), average closeness centrality (aCC), transitivity (Trans), average degree centrality (aDC), link density (LD), sMetric (sM) and graph energy (GE) of the constructed complex network) were compared on 5-min and UST segments to verify their validity and robustness in discriminating CH and CO under different data lengths. Besides, their potential for quantifying ANS-Load were also investigated.

**Results:** The validation results of HRV and VG features in discriminating CH from CO showed that VG features were more clearly distinguishable between the two groups than HRV features. For effectiveness evaluation of analyzing ANS on UST segment, the NNmean, SDNN, RMSSD, LF, HF and LF/HF in HRV features and the CC, Trans, Dia and GE of VG features remained stable in both activated and inactivated segments across all data lengths. The capability of HRV and VG features in quantifying ANS-Load were evaluated and compared under different ANS-Load, the results showed that most HRV features (SDNN, LFHF, RMSSD, vLF, LF and HF) and almost all VG features were correlated to sympathetic nerve activity intensity.

**Conclusions:** The proposed autonomic nervous activity analysis method based on VG and SKNA offers a new insight into ANS assessment in UST segments and ANS-Load quantification.

## 1 Introduction

The autonomic nerve system (ANS), composed of the sympathetic nervous system and the parasympathetic nervous system, plays an important role in regulating cardiovascular function and cerebrovascular function. Dysregulation of the ANS can affect the brain’s perception of various stressors, disrupt the adaptive capacity of homeostasis restoration, and ultimately increase the risk of stress-related disorders such as cardiac arrhythmia, hypertension, atherosclerosis, and stroke (Sternberg and Schaller, 2020). More recently, ANS modulation has been proposed as a promising therapeutic strategy for the management of autonomic dysfunction-related stroke (Mo et al., 2019). Therefore, ANS monitoring and analysis before the development of stress-related disorders is of paramount importance for improving the prognosis of patients with dysfunction-related stroke.

The most widely used clinical assessment method of sympathetic nerve activity (SNA) is evaluating end-organ responses to ANS physiological stimuli, such as tilt table testing, valsalva maneuver, plasma catecholamines, baroreflex sensitivity, thermoregulatory sweat test, and heart rate variation (HRV) (Thomas et al., 2019). Among these tests, HRV analysis is a widely accepted and implemented method to non-invasively and conveniently assess sympatho-vagal balance (Thomas et al., 2019). In general, HRV is generated and analyzed from the long-term electrocardiogram (ECG) waves, such as 24-h Holter, and its change can reflect the dynamic/trend of ANS activity over time (Bodapati et al., 2017; Schneider et al., 2018). It is reported that poststroke patients with raised SNA and low HRV are at higher risk for arrhythmias (atrial fibrillation, ventricular tachyarrhythmia) or other ECG changes (prolonged QT, inversed T wave) (Constantinescu et al., 2018). In addition, HRV is also used as a biomarker for classifying acquired brain injury patients and healthy controls (Galea et al., 2018). Meanwhile, multiple functional outcomes (cognitive functions, physical activity, and emotional expression) can be manifested in HRV (Forte et al., 2019; Sharma et al., 2019). Thus, HRV can not only serve as an indicator of cardiac function, but also reflect the central modulation capacity to stress (Yperzeele et al., 2015; Fyfe-Johnson et al., 2016; Kim et al., 2018). However, HRV quantifies ANS modulation at the sinoatrial level, which is difficult to generalize to cardiac patients with abnormal rhythms (atrial fibrillation, premature beats, etc.) (Zhao et al., 2020).

As a non-invasive and versatile SNA assessment method, skin sympathetic nerve activity (SKNA) has been applied to many clinical events (Doytchinova et al., 2017; Kusayama et al., 2020) and been proven to have the potential to predict sympathetic tone in many applications (i.e., acute myocardial infarction (He et al., 2020), neurologic recovery patients (Liu et al., 2021a), and sleep apnea (Kutkut et al., 2021)). To this juncture, several parameters have been derived from SKNA to quantify SNA. The average voltage of SKNA (aSKNA) is validated to be correlated with heart rate, and can be used as a biomarker for fitness level and efficacy of exercise training (Liu et al., 2021b). The burst numbers of SKNA (bSKNA) and variable value of SKNA (vSKNA) (Zhang et al., 2019) are higher in ventricular arrhythmia patients than in control groups, indicating SKNA can be used to predict the ventricular arrhythmogenesis recurrence. The envelope of SKNA (eSKNA) is extracted to depict the temporal pattern of SKNA, and the cross-comparison results between SKNA clustering groups and non-SKNA clustering groups demonstrate that eSKNA can act as a valid surrogate marker to classify ANS regulation ability in acute myocardial infarction patients (Liu et al., 2021a). Although these parameters can reflect the ANS changes by empirical threshold-based nerve bursts detection, the low signal-to-noise ratio of SKNA will lead to misjudgments (Xing et al., 2022a). In addition, the low amplitude SKNA signal (0.5–80 µV) is susceptible to noise, increasing the difficulty of extracting sympathetic-related information (Zhang et al., 2022). Therefore, more work is still needed to effectively analyze the autonomic nervous activity from SKNA, especially in real-time application.

Complex network is an emerging nonlinear dynamics analysis method for complex systems. It has been employed in a variety of physical and engineering systems: weather conditions (Fang et al., 2017), finance (Zhao et al., 2018), biomedical applications (Gao et al., 2021). Recently, several network-based approaches have been proposed to map time series into complex networks, such as visibility graph (Xu et al., 2018), recurrence plot (Eroglu et al., 2018), ordinal partition network (Santos et al., 2022). In particular, visibility graph (VG) is a simple and fast computational framework for us to bridge the gap between time series and complex networks, and it has been successfully implemented in different fields. Bhaduri and Ghosh (2016) studied cardiac dynamics during meditation through multi-fractal detrended fluctuation and RR interval-based VG, and they found that VG was superior to multi-fractal detrended fluctuation in reflecting physiological effects on subjects undergoing meditation. Gao et al. (2017) developed a time-dependent limited penetrable VG, and applied it to RR intervals for classifying heart states of healthy, congestive heart failure and atrial fibrillation. León et al. (2020) used HRV features and VG features derived from the heart rate time series for the prediction of late onset sepsis in preterm infants, the results showed that the VG features in HRV analysis were useful for sepsis prediction in newborns. From these studies, VG complex networks are often constructed from RR intervals for heart rate-related applications, while no work has focused on the application of VG analysis in evaluating ANS with SKNA.

In this study, an autonomic nervous activity analysis method was proposed based on VG complex network and SKNA. Based on previous studies (Naredi et al., 2000; Chun-jing et al., 2013), we hypothesized that SNA was elevated in patients with cerebral hemorrhage (CH). Therefore, we collected ECG and SKNA from CH patients and control group (CO), and compared the derived HRV and VG features to evaluate their effectiveness in distinguishing CH from CO. In addition, the ANS analysis performance of HRV and VG features on ultra-short-time (UST) segments were evaluated to verify their robustness under different data lengths. Finally, the correlations between HRV and VG features and ANS-Load were investigated under different data lengths to explore their potential for quantifying the intensity of SNA.

## 2 Methods

### 2.1 Data acquisition

The ECG and SKNA were recorded by a portable data acquisition device designed in our previous work (Xing et al., 2022b). It consists of low-noise analog-front-end (ADS1299, Texas Instruments, Dallas, TX) for bio-potential signal acquisition, a microcontroller (STM32L476, STMicroelectronics) for the management of the whole system, and a power management circuits (powered by a 3.7 V rechargeable lithium polymer battery). In order to reduce the system noise floor, a low-noise first-stage amplifier (INA128) was implemented with the ADS1299 chip. The clinical signals were measured at 4 kHz sampling frequency using conventional disposable silver/silver-chloride (Ag/AgCl) electrodes attached to the users’ chest. The signal measurements were carried out in a noise-free sound insulation room. After an adjustment period of at least 10-min, the 10-min signal of each subject was acquired in a supine position. The recorded signals were stored on a local trans-flash card, and processed off-line by MATLAB.

### 2.2 Patients

Patients with spontaneous CH who had a history of hypertension were recruited. All patients were male and had no definite cardiovascular and cerebrovascular events other than hypertension. The location of cerebral hemorrhage in all patients was located in the basal ganglia, and the hemorrhage did not break into the ventricle. The course of cerebral hemorrhage had passed through the acute phase and was in the subacute phase. Age- and sex-matched normal volunteers, no other obvious cardiovascular and cerebrovascular diseases except hypertension, were recruited as CO from the hypertension clinic. All the patients were enrolled from the Department of Neurosurgery, First Affiliated Hospital of Nanjing Medical University from October 2021 to December 2021. Exclusion criteria included: 1) patients with traumatic cerebral hemorrhage, ischemic stroke or hemorrhagic conversion; 2) cerebral hemorrhage patients underwent the unstable phase (with shock, large fluctuations in heart rate or blood pressure); 3) patients with thyroid disease, diabetes, cardiac arrhythmia, and other disorders that may affect ANS.

Sixteen patients were enrolled in this study, including seven CH patients and nine CO patients. A 10-min single-lead ECG and SKNA were recorded in a supine position for each patient, and they were asked to avoid unnecessary movement during the recording. Three Ag/AgCl electrodes were placed in the left subclavian, right subclavian, and right lower abdomen, and the sampling rate was 4 kHz.

### 2.3 Data process

#### 2.3.1 Signal pre-processing

Due to the small amplitudes of ECG and SKNA, the signal is easily contaminated by various noises. Therefore, the signal quality is visually assessed before signal processing. Those episodes that are corrupted by severe background noise and cannot distinguish QRS complexes are eliminated. Afterwards, only 5-min segments with more than 90% high signal quality are reserved, and the ECG and SKNA are extracted from these segments by 10th-order Butterworth bandpass filters with cutoff frequencies of 0.5–150 Hz and 500–1,000 Hz, respectively. For further HRV analysis, the QRS complexes are identified by P&T method (Pan and Tompkins, 1985), and false and missing detection are calibrated artificially. To clearly label neural clusters, eSKNA was extracted by performing moving average (MA) and root mean square (RMS) on SKNA (Eqs. 1, 2). Referring to (Liu et al., 2021a), the window size and sliding step of MA are 100-ms and 2-ms, respectively.

$$x_{\mathrm{MAk}}=\frac{\sum_{i=s*k}^{s*k+w}x_{i}}{m}$$ (1)

$$j=\frac{(Fs*T-w)*s}{Fs}$$ (2)

where $X_{\mathrm{MA}}$ is the array of input signal after MA; $x_{\mathrm{MAk}}$ is the *k*th sample of $X_{\mathrm{MA}}$ ; *j* is the number of $X_{\mathrm{MA}}$ ; *w* is the window size; *s* is the sliding step; $x_{i}$ is the *i*th sample of the input signal; *Fs* is the sampling frequency; *T* is the duration in second of selected data.

In RMS calculator (Eq. 3), the $X_{\mathrm{RMS}}$ is extracted from $X_{\mathrm{MA}}$ with a window size of 100-ms and a sliding step of 2-ms:

$$X_{\mathrm{RMS}}=\sqrt{\frac{\sum_{i=k}^{k+j}x_{\mathrm{MAi}}^{2}}{n}}$$ (3)

where *n* is the number of samples in a window; $x_{\mathrm{RMS}}$ is the *k*th sample value of RMS; *j* is the number of samples of $X_{\mathrm{RMS}}$ ; $X_{\mathrm{MAi}}$ is the *i*th sample value of the array $X_{\mathrm{RMS}}$ .

The $X_{\mathrm{RMS}}$ is defined as eSKNA, and a threshold-based method is performed on it for SKNA bursts determination. The threshold is calculated as follows:

Threshold=(Baseline−Min)∗5+Min (4)

where *Baseline* is the average of the lower 20% samples in the selected window; *Min* is the minimum of the selected window.

**FIGURE 1:**
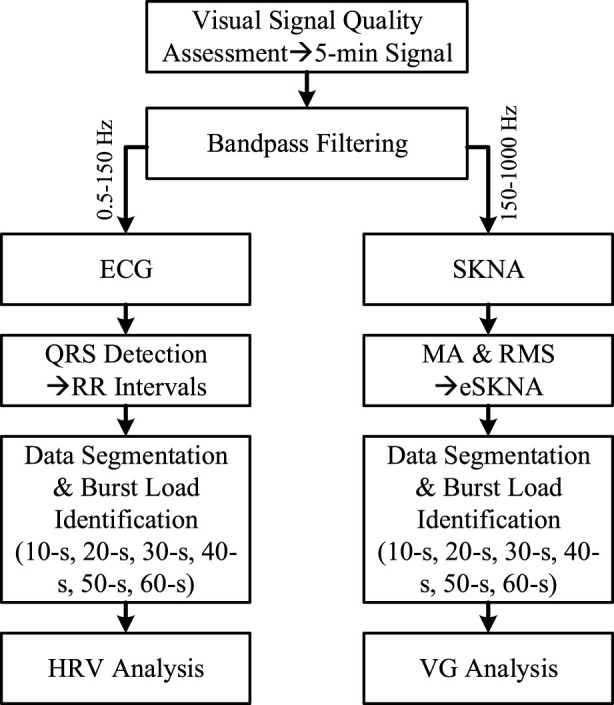
The flowchart of data process in this paper. MA and RMS mean the operation of moving average and root mean square, respectively.

In order to analyze the effectiveness of VG features in quantifying ANS from UST segments, the 5-min signals were split into 10-s, 20-s, 30-s, 40-s, 50-s and 60-s segments, respectively. The burst load of each segment was calculated as the ratio of burst time to total time, and partitioned to 5 equal intervals from 0 to 1 ([0, 0.2), [0.2, 0.4), [0.4, 0.6), [0.6, 0.8), [0.8, 1.0]). Then, the segments were marked as activated (burst load > 0) and inactivated (burst load = 0) according to the burst load. Thereafter, the HRV and VG analysis were conducted on these data. The flowchart of this paper is illustrated in Figure 1.

#### 2.3.2 Heart rate variation analysis

The time-domain features, frequency-domain features, and nonlinear features were extracted by the PhysioNet Cardiovascular Signal Toolbox (Vest et al., 2018). The time-domain analysis included sinus RR intervals-related features (mean (NNmean), standard deviation (SDNN), and root mean square difference (RMSSD)). The frequency-domain features consisted of the power in different frequency range (ultra-low frequency power (<0.003 Hz, uLF), very low frequency power ([0.003 Hz, 0.04 Hz), vLF), low frequency power ([0.04 Hz, 0.15 Hz), LF), high frequency power ([0.15 Hz, 0.4 Hz), HF), ratio of LF to HF (LFHF)). The nonlinear features consisted of the standard deviation of projection of the Poincaré Plot on the line perpendicular to the line of identity (y = −x, SD1), and approximate entropy (ApEn). The standard deviation of the projection of the Poincaré Plot on the line of identity (y = x, SD2) was not included in this study because it was not suitable for UST HRV analysis.

#### 2.3.3 Visibility graph features extraction of envelope of SKNA

As a natural graph-theoretical description of nonlinear systems, VG can simply convert a time series into a scale-free network. The statistical measures from the constructed network can demonstrate the dynamic behaviors of the nonlinear systems, and have been proven to be related to the self-similarity and complexity of the time series (Bhaduri and Ghosh, 2016). Every data point of time series is mapped to a node in its associated VG network, and an edge between two nodes is connected if the corresponding time samples can view each other. Suppose the original time series is *X*, and the *i*th point of *X* is *X*_*i*_. Two data points (*t*_*a*_, *X*_*a*_) and (*t*_*c*_, *X*_*c*_), at time *t*_*a*_ and *t*_*c*_, are connected if and only if any other data (*t*_*b*_, *X*_*b*_) between them (*t*_*a*_ < *t*_*b*_ < *t*_*c*_) satisfies the following criterion:

$$X_{b}<X_{c}+\left(X_{a}-X_{c}\right)*\frac{t_{c}-t_{b}}{t_{c}-t_{a}}$$ (5)

The VG network are applied on eSKNA, and ten network measures (Pan and Tompkins, 1985; Hou et al., 2016; Vest et al., 2018; Xing et al., 2022b; Santos et al., 2022) are extracted for further analysis in this study:

##### 2.3.3.1 Diameter

The longest shortest path between any two nodes in the network (Eq. 6).

$$\mathrm{Dia}=\mathrm{ma}x_{i,j}D_{\mathrm{ij}}$$ (6)

where *D*_*ij*_ is the length of the shortest path between node *i* and node *j*.

##### 2.3.3.2 Average node degree

The degree of a node in a graph is defined as the number of connected edges to this node, and the mean degree (Eq. 7) is the average value of all node’s degree in this graph (León et al., 2020).

$$\mathrm{aND}=\frac{1}{Ν}\sum_{n=1}^{N}d_{n}$$ (7)

where *N* is the total number of nodes, and *d*_*n*_ is the degree of node *n*.

##### 2.3.3.3 Average shortest-path length

The shortest path is a reflection of transmission and communication in the graph, the average shortest path length (Eq. 8) is the average of shortest path over all couples of nodes (Hou et al., 2016).

$$\mathrm{aSPL}=\frac{1}{\mathrm{Ν*}\left(\mathrm{Ν-1}\right)}\sum_{i,j\in V,i\neq j}D_{\mathrm{ij}}$$ (8)

where *N* is the total number of nodes, and *V* is the set of *N* nodes.

##### 2.3.3.4 Clustering coefficient

The cluster coefficient of a node in a graph is the ratio of all triangles involving that node to the number of connected triples centered on that node, and the cluster coefficient of a graph (Eq. 9) is the average of the cluster coefficient of all nodes (León et al., 2020).

$$\mathrm{CC}=\frac{1}{Ν}*\sum_{i\in V}\frac{e_{i}}{k_{i}*\left(k_{i}-1\right)}$$ (9)

where *e*_*i*_ is the actual number of edges between all the couples of neighbors of node *i*, and *k*_i_*(*k*_*i*_ *-*1) is the maximum possible number of edges between all the *k*_*i*_ neighbors of node *i*.

##### 2.3.3.5 Average closeness centrality

Closeness centrality is the sum of the distances from a node to other nodes, representing the convenience and ease of connection between the focal node and other nodes (Zhang and Luo, 2017).

$$\mathrm{aCC}=\frac{1}{Ν}\sum_{i=1}^{N}\frac{N-1}{\sum_{j=1}^{N}D_{ij}}$$ (10)

##### 2.3.3.6 Transitivity

The transitivity (Eq. 11) is the ratio between the triangle numbers and the connected triple numbers in a graph to obtain the global information of CC (León et al., 2020).

$$\mathrm{Trans=}\frac{\mathrm{3*number}\;\mathrm{of}\;\mathrm{trangles}\;\mathrm{in}\;\mathrm{the}\;\mathrm{graph}}{\mathrm{number}\;\mathrm{of}\;\mathrm{connected}\;\mathrm{triples}\;\mathrm{in}\;\mathrm{the}\;\mathrm{graph}}$$ (11)

##### 2.3.3.7 Average degree centrality

Degree centrality is defined as the ratio between the number of nodes connected to the current node, and the total number of all nodes in the network (Zhang and Luo, 2017).

$$\mathrm{aDC}=\frac{1}{Ν}\sum_{i=1}^{N}\frac{\sum_{j=1}^{N}e_{ij}}{N-1}$$ (12)

##### 2.3.3.8 Link density

Link density (Eq. 13) is the ratio between the number of edges and the maximum possible number of edges (N*(N-1)/2) (Liu et al., 2015).

$$\mathrm{LD}=\frac{\sum_{i=1}^{Ν}\sum_{j=1}^{N}e_{ij}}{N*\left(N-1\right)/2}$$ (13)

##### 2.3.3.9 sMetric

The sMetric (Eq. 14) is the sum of products of degrees across all edges (Li et al., 2005).

$$sM=\sum_{i,j=1}^{Ν}d_{i}*d_{j}$$ (14)

##### 2.3.3.10 Graph energy

Graph Energy (Eq. 15) is defined as the sum of the absolute values of the real components of the eigenvalues ( $\lambda_{i}$ ) of the graph (Balakrishnan, 2004).

$$GE=\sum_{i}\left|\lambda_{i}\right|$$ (15)

## 3 Experiments and results

### 3.1 Comparison of heart rate variation and visibility graph on autonomic nerve system analysis

**FIGURE 2:**
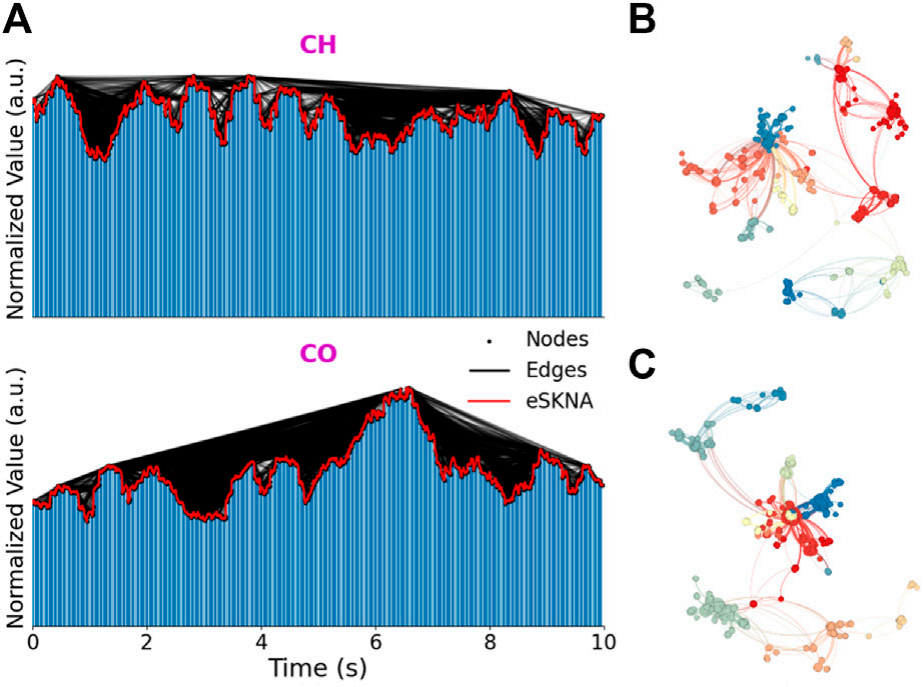
The typical eSKNA signals and their corresponding VG complex networks of CH and CO segments. **(A)** The typical eSKNA and their VG, **(B)** and **(C)** are the complex networks of CH and CO with colored communities.

The eSKNA segments are converted into a scale-free graph by natural VG method. The typical 10-s eSKNA of CH and CO segments are illustrated in Figure 2A, and their corresponding VG complex networks are shown in Figures 2B,C, respectively. For clear demonstration, the communities of these complex networks are colorized according to their modularity classes by Gephi software. It can be seen that the amplitudes of CH eSKNA fluctuate smoothly, while there is a clear burst in CO eSKNA around about 6-s time point (Figure 2A). The communities of the CH complex network are dispersed as all intermediate peaks obstruct the visible range between the front and rear peaks (Figure 2B). Conversely, the CO complex network consists of several small communities crowded with a central community, since the burst can view almost all other nodes in the network (Figure 2C).

**FIGURE 3:**
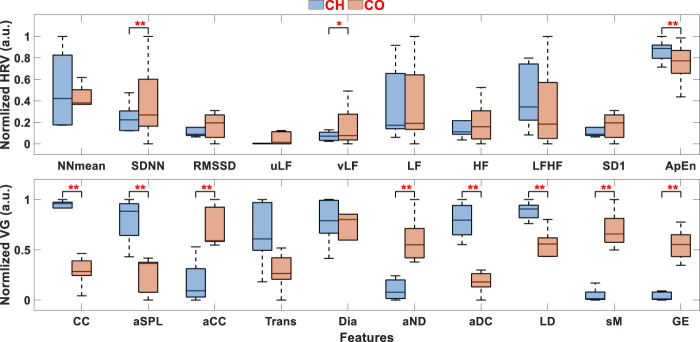
HRV and VG features between CH and CO. “*” stands for significant difference between two groups (*p* < 0.05) and “**” stands for extremely significant difference (*p* < 0.01). ANS Analysis Performance of VG on UST Segments.

To evaluate the validity of HRV and VG features from the 5-min signal, these features are normalized to [0, 1], and the comparisons between CH and CO for these features are illustrated in Figure 3. To further quantify the distribution differences between CH and CO, the WRS test is carried out for each feature. Significant difference (*p* < 0.05) between two groups is marked with red “*”, and extremely significant difference (*p* < 0.01) is marked with red “**“. In this paper, *p* values less than 0.05 were regarded as statistically significant for each test. Statistical analyses were performed using MATLAB (R2022a) on a PC with Intel® Core™ i7-7700 3.6 GHz processor and 32 GB RAM.

It can be seen from Figure 3, the distributions of most HRV features between CH and CO are overlapped, indicating the difficulty to distinguish CH from CO by these features. In contrast, the distribution differences between CH and CO are evident in most VG features, validating the effectiveness of VG features in classifying CH from CO. In addition, almost all *p*-values calculated from VG features are < 0.01 (except Trans and Dia), while only SDNN, vLF and ApEn show significant difference in HRV features.

The ANS function assessment by HRV analysis is typically performed on either 5-min ECG recordings or nominal 24-h recordings, which limits its application in dynamic conditions, such as dynamic sympathetic assessment in athletes. To investigate the ANS analysis performance of these parameters on UST signals, the 5-min signals are split to 10-s, 20-s, 30-s, 40-s, 50-s and 60-s segments, respectively. In addition, these segments are marked as ANS activated and inactivated depending to whether they contain ANS bursts labeled from eSKNA. Note that segments without valid QRS complexes are removed. The final number of each data length is illustrated in Table 1. The distribution of each feature among different data length is compared and quantified by KW test. Furthermore, the total runtime of VG feature extraction for each data length is compared.

**TABLE 1:** The number of data segments according to different data length, ANS status and burst load.

Data length (s)	Number of ANS status		Number of different burst load				
	Activated	Inactivated	[0, 20%)	[20%, 40%)	[40%, 60%)	[60%, 80%)	[80%, 100%]
10	335	136	310	111	39	8	3
20	162	65	154	52	15	5	1
30	107	44	104	30	14	2	1
40	77	29	73	24	8	1	0
50	63	26	59	25	4	1	0
60	56	23	50	24	4	1	0

**FIGURE 4:**
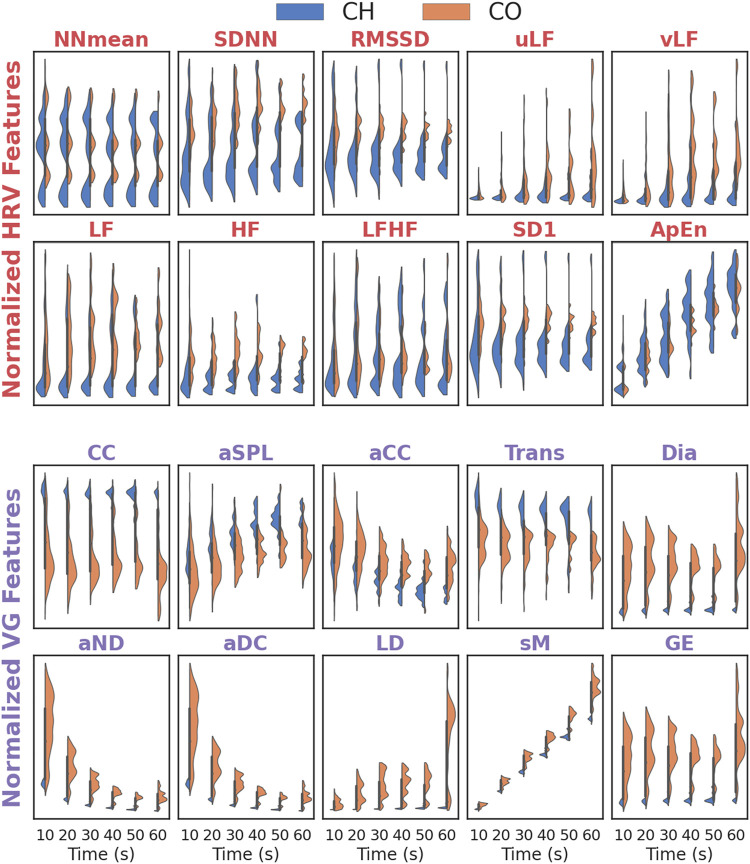
The distribution of features (HRV and VG) for different data length. Effectiveness of HRV and VG on ANS-Load Determination.

Figure 4 depicts the distribution differences of each feature (HRV and VG) for activated and inactivated segments under different data lengths. For almost all HRV features (except ApEn), the distribution for activated segments seldom changes with the data length increase, and the distribution for inactivated segments varies sightly in uLF, vLF and SD1. The ApEn for both activated and inactivated segments increases with data length expands. For VG features, the distributions for both activated and inactivated segments remain stable in CC, Trans, Dia and GE, and change slightly in aSPL and aCC. However, they decrease (increase) sharply with the data length increases in aND and aDC (sM).

To quantitatively characterize the stability of features (HRV and VG) in UST segments, their distribution differences (for activated and inactivated segments, respectively) in different data lengths (10-s, 20-s, 30-s, 40-s, 50-s and 60-s) are compared by KW test. The results are shown in Table 2. It is clear that the *p*-values of NNmean, SDNN, RMSSD, LF, HF, LFHF and SD1 are > 0.05 in both activated and inactivated segments, indicating that these features are not distributed differently across segments with different length. Similarly, the VG features of CC, Trans, Dia and GE also show no distribution differences in both activated and inactivated segments, as they all had *p*-values > 0.05. However, the *p*-values for vLF and ApEn of HRV features and aND, aDC, LD and sM of VG features are all < 0.01 for both groups, implying that their distribution varies significantly during data length increase.

**TABLE 2:** The KW test results of feature distribution differences in different data lengths.

	Status	Statistics	NNmean	SDNN	RMSSD	uLF	vLF	LF	HF	LFHF	SD1	ApEn
HRV	Activated	Chi-sq	0.17	5.64	0.55	11.04	27.52	0.89	0.70	0.57	0.42	70.51
		p	0.920	0.059	0.761	0.004	0.000	0.641	0.703	0.754	0.810	0.000
	Inactivated	Chi-sq	0.27	5.62	0.88	4.14	18.14	1.67	1.09	0.58	0.65	153.27
		p	0.876	0.060	0.645	0.126	0.000	0.434	0.579	0.750	0.721	0.000
VG	Status	Statistics	CC	aSPL	aCC	Trans	Dia	aND	aDC	LD	sM	GE
	Activated	Chi-sq	0.13	3.77	3.65	2.16	0.27	43.31	43.31	15.22	68.23	0.27
		p	0.937	0.152	0.161	0.339	0.874	0.000	0.000	0.000	0.000	0.874
	Inactivated	Chi-sq	0.49	6.85	6.45	2.60	1.23	37.44	37.44	19.58	157.94	1.23
		p	0.785	0.033	0.040	0.272	0.540	0.000	0.000	0.000	0.000	0.540

**FIGURE 5:**
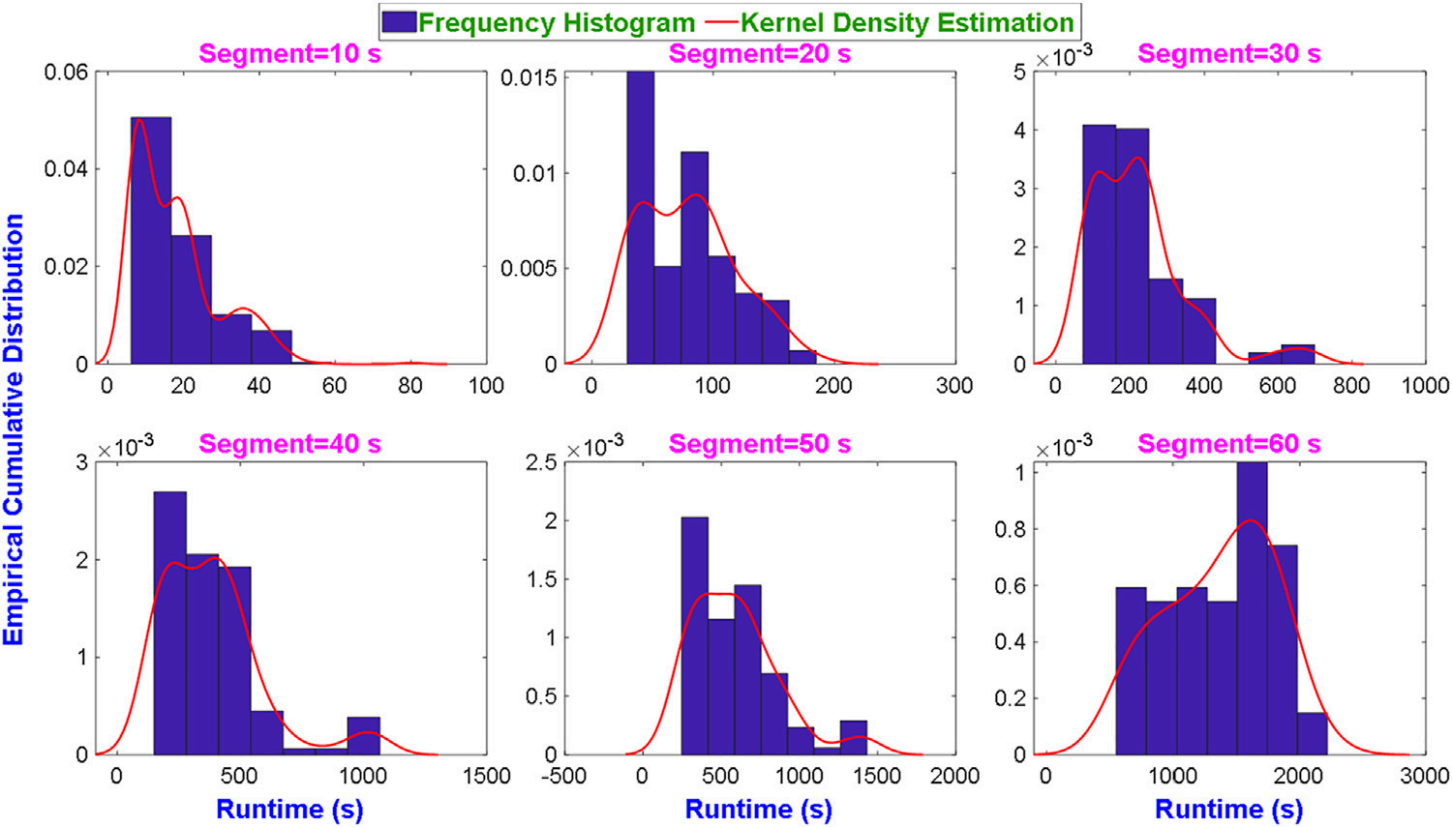
The running times of VG feature extraction under different data lengths.

Although several features (HRV and VG) show stable performance in short-term segments, their efficiency still needs to be investigated to ensure their practical application. As we all known that the computational complexity of HRV features is very low, therefore, we only compare the running time of VG features under different data lengths. Figure 5 shows the histogram based on empirical cumulative distribution function and kernel density estimation of the running time for VG features extraction from different data lengths. The distribution is heavy-tailed in 10-s and 30-s segments, but appears approximately normal distribution in the remaining segments. The average time for each data length is around 15-s, 80-s, 210-s, 360-s, 565-s and 1,420-s, respectively. Obviously, the average runtime increases rapidly with data length expands and shows an exponential growth trend. The reason is that the nodes of the VG complex network increase with data length, resulting in a rapid growth of computational complexity for extracting features from the constructed adjacent matrix.

In order to evaluate the capability of HRV and VG features in quantifying ANS-Load, the burst of each segment is determined by a threshold-based method. Furthermore, the burst load (ANS-Load) is extracted by calculating the ratio of the burst time to total time of the segment. The burst load is coarse-grained to 5 equal partitions from 0 to 1, the segment number of each partition under different data lengths is listed in Table 1. Then, the correlations between features and segment length under different burst load are quantified by Kendall rank correlation coefficient. As there are not enough ANS-Load in partitions [0.6, 0.8) and [0.8, 1.0], we only compare the correlation coefficient in ANS-Load among (0, 0.6).

**FIGURE 6:**
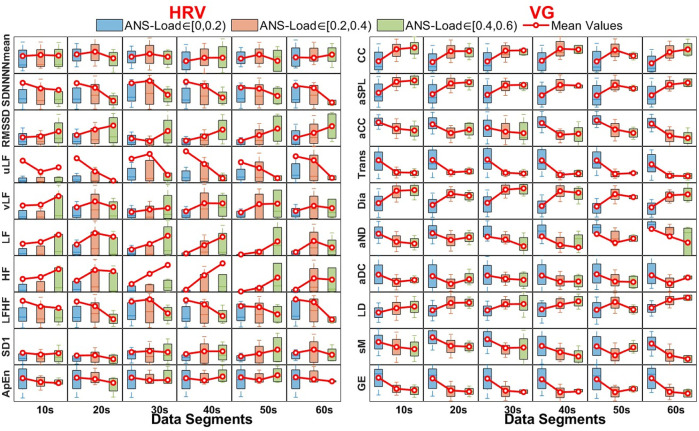
The distributions between each HRV and VG features and data lengths under different ANS-Load and their corresponding mean values.

The distributions between each HRV and VG feature and data lengths under different ANS-Load, associated with their mean values, are shown in Figure 6. In HRV features, the SDNN and LFHF (RMSSD, vLF, LF and HF) decrease (increase) with the ANS-Load increase in different data lengths, implying that the variation of ANS-Load would influence the time-domain and frequency-domain features of HRV. Meanwhile, almost all the VG features present an increasing or decreasing trend with the ANS-Load increase. The reason may be that the increased autonomic activity is reflected in increased bursts in eSKNA, resulting in the variation of connections between two nodes.

**FIGURE 7:**
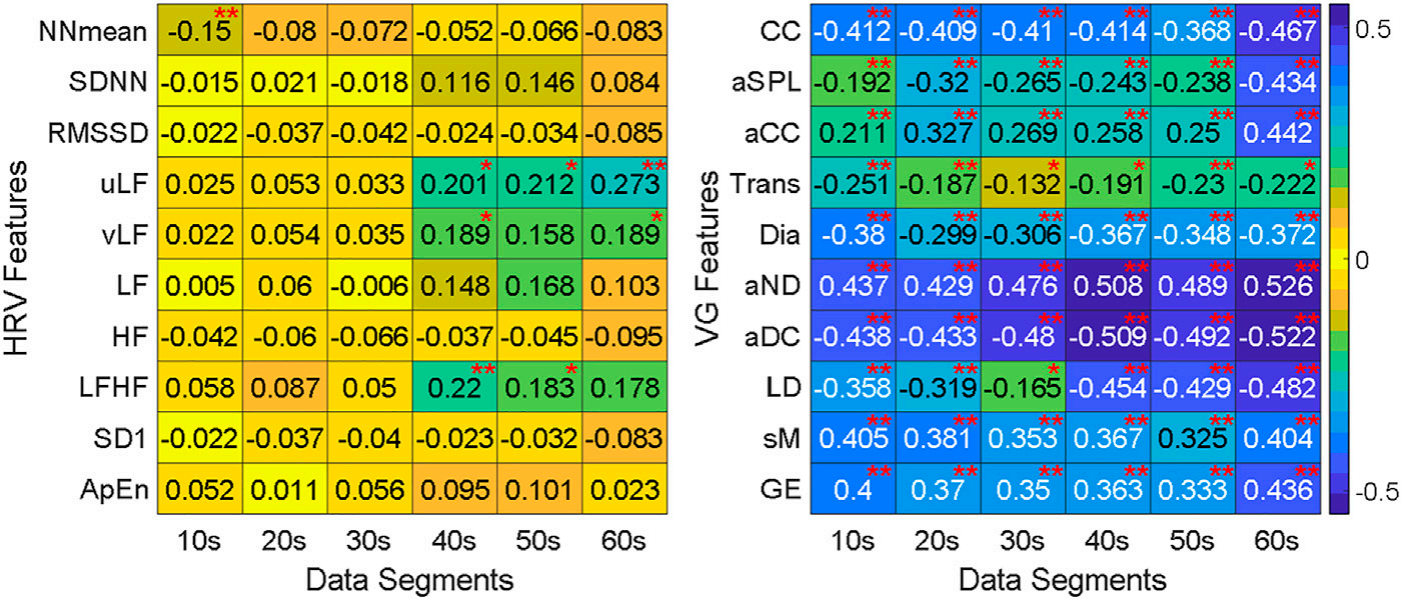
The Kendall rank correlation coefficients between features and segment length under different burst load. “*” stands for significant correlation between features and ANS-Load (*p* < 0.05) and “**” stands for extremely significant correlation (*p* < 0.01).

The Kendall rank correlation coefficients for HRV and VG features are illustrated in Figure 7, and the red “*” and “**” represent *p*-values < 0.05 and < 0.01, respectively. In this study, we only focus on the degree of correlation other than its direction, therefore the positive correlation and negative correlation share the same color in Figure 7. It is obvious that there is a weak correlation between HRV features and ANS-Load, most correlation coefficients are around 0, and the maximum is 0.273 for uLF in 60-s segment. On the contrary, the VG features show a stronger correlation with ANS-Load, especially the correlation coefficient of aND reaches 0.526 in 60-s. In addition, the absolute values of the correlation coefficients are all above 0.13. Besides, only few HRV features show significant correlation between features and ANS-Load (i.e., NNmean in 10-s, LFHF in 20-s, vLF in 40-s). However, almost all the correlations between VG features and ANS-Load are extremely significant, indicating the potential of VG features for ANS-Load quantification.

## 4 Discussion

A VG and SKNA based autonomic nervous activity analysis method was proposed in this paper. SKNA overcomes the sinoatrial level limitations of traditional HRV analysis, and a number of SKNA-derived metrics had been proposed for SNA quantification. However, the noise-susceptibility of these metrics required new methods for quantitatively ANS assessment, especially for short-time segment application. As a nonlinear analysis method, VG offered a new insight into ANS assessment in short-term segments and ANS-Load quantification.

The unique contribution of this paper was the first application of VG on eSKNA for ANS assessment. HRV was the most widely used ANS assessment method, and its indices from time-domain, frequency-domain and nonlinear-domain had been proved to be biomarkers for cardiac arrhythmias, brain injury and emotion (Fyfe-Johnson et al., 2016; Constantinescu et al., 2018; Galea et al., 2018). However, HRV from UST data could not fully show the nerve activity. The newly proposed noninvasive cardiac SNA assessment method (SKNA) and its derived metrices has been widely used in clinical events, such as acute myocardial infarction (He et al., 2020), neurologic recovery patients (Liu et al., 2021a), and sleep apnea (Kutkut et al., 2021). Nevertheless, these SKNA-derived metrics could only qualitatively analyze ANS and could not quantitatively reflect ANS intensity or ANS-Load. Thanks to the nonlinear dynamics analysis method–VG complex networks, we could evaluate the ANS from network aspect. Although many previous studies had investigated and compared VG and HRV in meditation analysis (Bhaduri and Ghosh, 2016), sleep assessment (Hou et al., 2016) and congestive heart failure (Gao et al., 2017), this paper was the first to employ VG on eSKNA for ANS assessment. The comparison of HRV and VG features between CH and CO (Figure 2) showed that VG features are superior to HRV features in the ANS analysis. There were no significant distribution differences between CH and CO for most HRV features, while almost all VG features were clearly distinguishable between the two groups.

The stability of HRV and VG features in UST segments were compared by quantifying their distribution differences against different data lengths. Most HRV features remained stable for both activated and inactivated segments under all data lengths. In addition, the time-domain features (NNmean, SDNN, RMSSD) and most frequency-domain features (LF, HF, LFHF) manifested conformity in these segments, indicating that most UST HRV features could be used as surrogates for short-term HRV features. These results were consistent with Castald’s (Castaldo et al., 2019) investigation that NNmean and HF displayed consistency across all of the excerpt lengths (30 s, 1 min, 2 min, 3 min, and 5 min) for mental stress assessment. However, Jin Woong et al. (Kim et al., 2021) studied UST HRV in non-static conditions by comparing UST HRV features (10, 30, 60, 120, 180, and 240-s) with those from 5-min HRV, the results showed that UST HRV variables derived from the static condition could not applied to the non-static conditions of daily life. Similarly, the CC, Trans, Dia and GE of VG features remained stable in both activated and inactivated segments across all data lengths, implying that these VG features could reveal the dynamical changes caused by the adjustment of autonomous neural system from UST segments. Likewise, Jiang et al. (2013) applied VG to heartbeat interval time series for meditation investigation, and they also tested the stability of VG features on different length data, the results showed that the data length had no prominent effect on the VG analysis. The reason may be that the degree distribution persisted the same form for different length of data in any activated and inactivated segments.

HRV had been used as a biomarker for SNA measurement, but seldom been used for quantifying ANS-Load. The correlations between HRV features and ANS-Load were studied at different data lengths, and the comparison results showed that time-domain and frequency-domain features (SDNN, LFHF, RMSSD, vLF, LF and HF) had the potential to quantify ANS in UST segments. From a multimodal perspective, Debnath et al. (2021) designed a template matching algorithm to calculate scaled and stretched HRV features, associated with other features, for sympathetic and parasympathetic parameters determination. However, the acquisition of these employed features was complicated, and it still required more other biomarkers or calculated features to improve the quantification accuracy for practical clinical applications. The SKNA had been applied to evaluate the ANS as a non-invasive method in many clinical applications (Zhang et al., 2022), and many SKNA-derived metrices (e.g., aSKNA, bSKNA) had been validated and used for ANS qualification. Nevertheless, these parameters were susceptible to noise. In this paper, the VG features on eSKNA were extracted and compared across different ANS-Load, the results showed that almost all the VG features were correlated to ANS-Load. The link-related features (CC, aPL, Dia and LD) increased as ANS-Load grow, while the degree-related features (aCC, Trans, aND, aDC and sM) presented a decreasing trend across increasing ANS-Load. The increase in SNA intensity was manifested as the rising number and duration of bursts in eSKNA, which leads to a growth in the possibility of links between any two nodes in the VG. However, these links only concentrated on certain nodes (peak points of bursts), it meant that the node degrees of the entire VG would be aggregated into these nodes, resulting the increase of community numbers and the decrease of average degree. In addition, the total number of links grow exponentially with the total number of nodes in the network, while the degree distribution did not change with the node numbers (Tessone et al., 2011).

One limitation of our study is the small number of participants, further studies with larger cohorts are needed to confirm and strengthen these results. Another limitation is the VG features are only compared with HRV features, its validity still needs comparison with demographic information and laboratory tests for practical clinical applications. In addition, the robustness against noise of this method needs more efforts.

## 5 Conclusion

In summary, a VG on eSKNA based autonomic nervous activity analysis method was proposed in this paper. The comparison results of the HRV and VG features on CH and CO segments showed the superiority of VG features in ANS analysis. Furthermore, the ANS analysis performance of VG features on eSKNA signals with different data lengths demonstrated the stability of VG features (aND, aDC, LD and sM) in discriminating activated and inactivated segments at different data lengths. In addition, the capability of HRV and VG features to quantify SNA intensity was also evaluated, and the results showed that VG features had the potential to determine ANS-Load.

## Data Availability

The raw data supporting the conclusions of this article will be made available by the authors, without undue reservation.
